# Microbial Lifestyle and Genome Signatures

**DOI:** 10.2174/138920212799860698

**Published:** 2012-04

**Authors:** Chitra Dutta, Sandip Paul

**Affiliations:** Structural Biology & Bioinformatics Division, CSIR- Indian Institute of Chemical Biology, 4, Raja S. C. Mullick Road, Kolkata 700032, India

**Keywords:** Reductive genome evolution, Intra-amoeba pathogens, Strand-specific codon bias, Genome islands, Trophic strategies, Thermophiles, Halophiles.

## Abstract

Microbes are known for their unique ability to adapt to varying lifestyle and environment, even to the extreme or adverse ones. The genomic architecture of a microbe may bear the signatures not only of its phylogenetic position, but also of the kind of lifestyle to which it is adapted. The present review aims to provide an account of the specific genome signatures observed in microbes acclimatized to distinct lifestyles or ecological niches. Niche-specific signatures identified at different levels of microbial genome organization like base composition, GC-skew, purine-pyrimidine ratio, dinucleotide abundance, codon bias, oligonucleotide composition etc. have been discussed. Among the specific cases highlighted in the review are the phenomena of genome shrinkage in obligatory host-restricted microbes, genome expansion in strictly intra-amoebal pathogens, strand-specific codon usage in intracellular species, acquisition of genome islands in pathogenic or symbiotic organisms, discriminatory genomic traits of marine microbes with distinct trophic strategies, and conspicuous sequence features of certain extremophiles like those adapted to high temperature or high salinity.

## INTRODUCTION

Micorbes are the most ancient and tiny, yet the most diverse and versatile life forms of our planet! For nearly four billion years, they have evolved to adapt themselves to every lifestyle imaginable and every environment conceivable, including the most extreme and inhospitable ones. The genome architectures of microorganisms often bear the telltale signs of this long journey of adaptive evolution. Microbes from distant lineages but of similar lifestyle may exhibit similar genomic/proteomic traits, telling the tale of tailor-made convergence. On contrary, closely related bacterial species, even strains of the same species, when acclimatize to distinct ecology, may display substantial genomic diversity, narrating the history of niche-driven divergence. The lifestyle of a microbe, therefore, not only can contribute significantly in sculpting its genome, but also may inscribe own signature in its genome fabrics. The present review aims to provide an account of such niche-specific genome signatures in microorganisms adapted to specialized lifestyle and/or environment.

The term “genome signature”, coined by Karlin & Burge [1], has been used by various investigators to refer to similar concepts, but to different genomic properties. Typically, a genome signature refers to any sequence feature that enables characterization of the source organism from mere knowledge of its nucleotide sequence (complete or even partial genome sequence of sufficient length). The major advantage of the concept of genome signatures over the traditional approaches of rRNA-based phylogeny is that it does not depend on sequence alignment [2-4]. An ideal genome signature should satisfy two major criteria – i) it should be species-specific, *i.e.*, the signature should be different for different genomes and ii) it should be pervasive, *i.e.*, the imprint of the global signature should persist locally at smaller scales throughout the genome. Signatures of closely related species are expected to be more similar to one another than the signatures of distant ones. Usually “closely related species” refers to close phylogenetic lineages. However, the present review intends to focus on the signatures of “ecological kinship” rather than those of the “taxonomical cliques”.

## SEQUENCE FEATURES OF MICROBIAL GENOMES INFLUENCED BY LIFESTYLE

### a) G+C-Content

The simplest compositional parameter that might be influenced by environment or lifestyle of a microbial species is the G+C-content of its genome [5-7], which remains fairly constant within a microbial species, but varies widely across microbial species. The genomic G+C-content of a microbe, reflecting optimization between the directional mutational bias [8, 9], natural selection and genetic drift [10, 11], is often influenced by factors like temperature [12], niche complexity [5], cost and availability of nucleotides [13, 14], aerobiosis [15], nitrogen utilization [16] etc. There is a general tendency of large genomes to be G+C rich and small genomes to be G+C poor [17-19]. The obligatory intracellular pathogens/symbionts and microorganisms surviving in nutrient-limiting environments are, in most cases, characterized by relatively small genomes of low G+C-content, apparently in attempt to reduce replication expenses [13, 14], while free-living organisms, especially of the ones surviving in the soil [6], usually possess much larger genomes of higher G+C-content.

### b) Oligonucleotide Composition

Oligonucleotide frequencies capture species-specific characteristics of nucleotide composition more effectively than simple G+C-content [20]. Comparison of di-, tri- or higher order oligonucleotide frequencies in DNA sequences has long been used as a method of sequence characterization, particularly because this approach did not require sequence alignment [2-4]. The concept of a non-alignment, genomic signature approach to genome analysis was introduced for the first time by Karlin & Burge [1], when they defined “Dinucleotide relative abundances”, the deviation of observed dinucleotide frequencies from those expected from the mononucleotide frequencies. A series of work, conducted by Karlin & Burge as well as other groups of investigators [1, 21-23], have established the set of dinucleotide relative abundances as a robust genome signature that can discriminate between sequences from different organisms. Dinucleotide relative abundance values appear to reflect the chemistry of dinucleotide stacking energies and base-step conformational preferences, as well as the species-specific properties of DNA modification, replication and repair mechanisms [1, 22].

Karlin & Burge [1] speculated that dinucleotide genome signatures might be influenced by environmental influences such as pH, temperature and salinity. In a study of seven complete and several partial microbial genomes, Karlin *et al.* [22] noted that the dinucleotide TA, though broadly underrepresented in prokaryotes and eukaryotes, occurs with normal frequencies in two archaeal extremophiles - Sulfolobus and *Pyrobaculum aerophilum* and the dinucleotide CG is underrepresented in three thermophilic archaea, namely *Methanococcus jannaschii, Sulfolobus sp.,* and *M. thermoautotrophicum*, but overrepresented in halobacteria. It has later been reported [24] that the halophilic microbes, characterized by overrepresentation of the dinucleotides GA/TC, CG and AC/GT, can be differentiated from non-halophiles on the basis of their dinucleotide abundance values.

Parallel to the applications of dinucleotide relative abundance, oligonucleotides of varying length ranging from dinucleotides to octanucleotides have also been employed in combination with various metrics, clustering algorithms, or supervised machine learning methods to detect species-specific patterns in genome sequences from all kingdoms of life [20, 22, 25-34]. These signatures could not only detect taxonomic relationships, but also showed potential in delineating niche-specific patterns. Karlin *et al.* [22] reported that the tetranucleotide CTAG is extremely underrepresented and distributed in an anomalous fashion along the genome of the thermophilic microbe *M. jannaschi*. Applying classification and regression tree (CART) analysis to genome-wide tetranucleotide frequencies of 195 archaea and bacteria, Dyer *et al.* [35] reported the discriminating tetramers, the frequencies of which could differentiate between three temperature ranges, hyperthermophily, thermophily and mesophily.

Analysis of dinucleotide composition of bacteriophage genomes revealed that the phage genomes often display distinct genomic signatures depending on their replication and repair mechanisms [36]. The signatures of temperate phages, whose replication and repair depends on the host machinery, converge toward the signatures of their respective hosts, whereas autonomously replicating phages like T4 or T7 display their own characteristic signatures. Recently, use of tetranucleotide-based genome signatures enabled differentiation of the phages infecting *E. coli, S. aureus, M. smegmatis *and *P. aeruginosa*, where most of the temperate phages exhibited a shorter genomic signature distance between their genomes and that of their hosts than that of the lytic phages [37]. These observations advocate for the hypothesis that the intrinsic replication and repair mechanisms contribute significantly to the species-specific nature of dinucleotide relative abundances [38].

Free-living bacteria, in general, display stronger bias in oligonucleotide usage than host-associated bacteria, as observed in a hierarchical clustering based on hexanucleotide–based genome signatures of 867 prokaryotic genomes [31]. Recently, comparative analysis of tetranucleotide composition in a set of 774 sequenced microbial genomes revealed convergence of compositional patterns among genomes with similar habitats [34], displaying distinct clusters of obligate intracellular organisms (both pathogen and endosymbiont) and grouping of the halophilic bacterium *Salinibacter rubber*, not with its fellow Bacteroidetes, but with halophilic and methanogenic Archaea [34].

### c) Codon Usage

Trends in codon usage in microorganisms often carry the signals of their lifestyle or environment. Synonymous codon usage patterns in unicellular organisms, in general, follow species-specific biases that reflect an optimization between mutational biases and selective forces [39-42]. Among the major selection forces, translational selection is operative on large number of organisms, in which the highly expressed genes prefer to use a subset of synonymous codons [42]. The study conducted by Rocha [43] suggested that the fast-growing bacteria have higher codon usage bias in highly expressed genes due to the presence of fewer anticodons and hence, fewer subsets of distinct tRNAs. Lynn *et al.* [44] reported the presence of a characteristic pattern of codon usage among the thermophiles, which has later been reconfirmed by several investigators [45-48]. Distinct niche-specific trends in synonymous codon usage have also been observed in microbes thriving at high salinity [24]. An analysis of synonymous codon usage patterns in bacterial and fungal genomes by Willenbrok *et al.* [49] demonstrated that differences in codon preferences of translational codon adaptation and dominant codon adaptation provide an environmental signature that can segregate bacteria according to their lifestyle, for instance soil bacteria and soil symbionts, spore formers, enteric bacteria, aquatic bacteria, and small intercellular and extracellular pathogens.

Codon usage bias in viral genomes often reflects imprints of adaptation to specific host environment. The dinucleotide CpG and the CpG-containing codons are often significantly underrepresented in ORFs of small vertebrate DNA viruses [50], such as poliovirus genomes, especially in vaccine-derived poliviruses and the attenuated virus of polioviruses genotype 1 [51]. This might be due to the fact that the unmethylated CpGs are recognized by the host's innate immune system (Toll-like receptor 9) as a pathogen signature [52], while methylated CpGs in a small vertebrate DNA or RNA virus would face a high chance of mutation that would result in a reduction of this dinucleotide [50, 53]. A lower frequency of CpG might also help the vaccine derived polio virus out of the host immunity [51]. Multivariate analysis of codon usage patterns in the genes from segment 1 to segment 6 of avian and human influenza viruses, including pandemic H1N1, showed that the codon preferences of seasonal human influenza viruses were distinct among their subtypes and different from those of avian viruses [54] and a plausible explanation could be that the replication of the influenza virus depends on its host's machinery, and hence, the codon usage of the viral genes might be subject to host selection pressures, especially after interspecies transmission.

Apart from these factors, there are various other sequence attributes like purine-loading [55], GC-skew [56], genomic islands [57] etc, that are often employed to reveal specific evolutionary traits, which will be discussed later in relevant sections. Properties like conserved sequence repeats [58], “periodicity signatures” – the formal representation of periodic sequence patterns related to DNA curvature [59] and compositional spectra based on imperfect occurrences of long olignucleotide words [60, 61] are also potentially characteristic of different ecological groups of microbes. For instance, the archaea of the order Halobacteriaceae displayed the “periodicity signatures” distinct from other archaeal species, which might be due to their early divergence from other archaeal lineages, extensive lateral gene transfer or adaptation to high salt environments [59]. Clustering of genomes of 39 species of Eukarya, Eubacteria, and Archaea using the compositional spectra [61] could classify the organisms on the basis of two ecological parameters, temperature and oxygen.

## SPECIALIZED LIFESTYLES OF MICROBIAL COMMUNITY & THEIR GENOME SIGNATURES

### a) Obligatory Intracellular Lifestyle, Characterized by Genome Reduction 

Bacteria often trade their free-living lifestyle for an obligatory symbiotic or parasitic relationship with eukaryotic hosts. Examples include endocellular symbionts like *Wigglesworthia glossinidia *or *Buchnera aphidicola*, as well as pathogenic bacteria like *Mycobacterium leprae*, the causative agents of leprosy, *Borrelia burgdorferi,* the agent of Lyme disease and many other parasitic bacteria. Such host-restricted bacteria usually display some specific genome features, not observed in their close relatives retaining free-living stages [18, 62-65]. These include much smaller genome sizes; significant reduction in gene repertoire; accumulation of pseudogenes; accelerated sequence evolution; appreciable enrichment in A+T-content; and significant increase in the frequency of mobile elements in the genomes, in some cases [62, 63, 66]. All these features together represent a general syndrome of reductive genome evolution, which has been observed repeatedly in obligatory intracellular microbes from diverse lineages and of distinct host environment.

There could be various factors driving reductive evolution in host-restricted bacteria. Within the protected and predictable environment inside the host, many genes are rendered redundant or superfluous, and previously deleterious mutations become neutral in effect, due to relaxed selection. Furthermore, an obligatory association with host may result in the drastic reduction in effective population size of a lineage owing to the strict vertical inheritance modes, thereby leading to recurrent bottlenecks [66-70] and these may lead to an increase in the fixation rates for slightly deleterious mutations [71], as postulated by the so-called Muller’s ratchet [72]. Limited opportunities for the horizontal transmission of genetic elements in the secluded lifestyle of endosymbionts are also likely to contribute to smaller genome sizes. One could argue that genome shrinkage represents a selective process of genome streamlining, by which the organisms judiciously get rid off extraneous DNA. But the retention of nonfunctional DNA in the form of pseudogenes or increase in spacer regions in the reduced genomes, as observed in *Rickettsia* or *Buchnera* [62], advocates for the notion of the genetic drift, rather that of the selection driven genome shrinkage.

Intracellular microbes, in general, follow some common trends for gene retention/disposal. Small genomes, in general, retain fewer tRNAs and fewer DNA repair/recombination enzymes [73]. Relatively A+T–rich genomes of host-restricted organisms may be attributed, at least partially, to the elimination or decreased efficiency of genes encoding DNA repair enzymes [74]. For instance, in *U. urealyticum*, the mutation pressure leading to A+T enrichment may be attributed to the decreased ability to remove uracil from DNA due to the absence/inefficiency of the DNA repair enzymes dUTPase, which prevents dUTP from being integrated into DNA, and uracil-DNA glycosylase, which removes uracil from DNA. Spontaneous deamination of deoxycytidine and mis-incorporation of dUTP by DNA polymerase are the sources of uracil in DNA, and simultaneous repair of GU mismatches by DNA polymerase leads to an A/T enrichment [74]. Genes involved in redundant/unneeded pathways like biosynthesis pathways, transcriptional regulatory mechanisms or regulatory elements like sigma factors [65, 73] are usually eliminated, while the genes involved in essential functions like DNA replication, transcription and translation, chaperone systems and the protein translocation machinery are likely to be retained. As revealed in *Buchnera* [75], reductive genome evolution may lead to a shrinkage in the modular structure of their protein interaction networks in a way to maintain the essential characteristics of the networks. Symbiont lineages often retain distinctive gene sets, depending on their provisioning roles in hosts, as observed in *Buchnera,* *Wigglesworthia* and *Blochmannia* [76].

Newly host-dependent bacteria, which are still in the process of transition from free-living lifestyle to obligatory host association may be distinguished from anciently host-restricted ones by two genomic attributes: expansion of insertion sequence (IS) elements and abundance of pseudogenes. Recently evolved endosymbionts/pathogens that are still in transition usually possess much higher numbers of IS elements as compared to their free-living relatives. At initial stages of host restriction, IS elements can promote genome degradation by inactivation of genes [77] and regulatory elements [78], and also by serving as repetitive sequences that induce large deletions through homologous recombination [79]. The anciently host-restricted genomes might also have passed through such stages of IS spread, but traces of these mobile elements have now been deleted or mutated beyond recognition, as exemplified by the extreme genomic stasis of *Buchnera* having no sign of chromosome rearrangements or gene acquisitions in the past 50 to 70 million years [80]. One striking exception is *Wolbachia*, which, despite carrying typical features of anciently host-restricted bacteria, retains very large numbers of mobile elements [81]. It has been suggested that abundance of mobile elements might enable this arthropod-associated endosymbiont to coinfect individual insect hosts and undergo lineage-specific gene rearrangements.

Another distinctive signs of ongoing gene inactivation in genomes of host-restricted bacteria is the presence of numerous pseudogenes, observed in *Rickettsia prowazekii *[82], *M*. *leprae *[83] and many other microbes. In a genome under the process of shrinkage, inactivation of individual genes results in pseudogenes that slowly dwindle through deletions and as a result, ancestral genes are present in varying stages of decline [62].

It has recently been suggested that the phenomenon of reductive evolution could be a distinct characteristics of the bacteria specifically associated with human communities, agriculture and animal domestication - three features clearly linked to the Neolithic revolution [84]. It is hypothesized that after the first Neolithic settlements, bacteria specialized in human-associated niches underwent the reductive evolution, which did not occur in related species that are not specialized in humans. Recently, a comparative study of genome evolution in *Lactobacillus reuteri* populations associated with rodent and human hosts revealed that the rodent-restricted strains possess a large and adaptable pan-genome while its human-restricted relatives are subjected to a process of reductive evolution [85].

### b) Non-Specialized Intra-Amoebal Lifestyle, Characterized by Genome Expansion 

The notion of post-neolithic genome reduction in human-associated intracellular bacteria has also been supported by the observation that intra-amoebal pathogens exhibit, in general, increased genome size compared to their human-specialized relatives [86]. Free-living amoebae feed on several bacteria, fungi, and algae that they encounter. Some microorganisms, which have evolved to resist these phagocytic protests, survive and replicate within their amoebal host. These amoeba-resistant microorganisms include many established pathogens like *Legionella spp., Chlamydophila pneumoniae, Mycobacterium avium, Listeria monocytogenes, Pseudomonas aeruginosa* etc. Interestingly enough, many of these amoeba-resistant pathogens, despite their strict intracellular lifestyle, are reported to have larger genomes as compared to their human-infecting relatives [86]. It has been proposed that these nonspecialized microorganisms live in community within their hosts, promoting horizontal gene exchanges between different sympatric intra-amoebal parasites, as well as between the parasites and amoeba, which increases their genome sizes [86, 87].

### c) Strand-Specific Codon Bias, Frequently Observed in Intracellular Microorganisms

Another distinctive genomic feature, observed in a number of obligate intracellular bacteria is significantly different synonymous and/or non-synonymous codon usage patterns in genes transcribed on the leading and lagging strands of replication [88, 89]. Bacterial genomes are, in general, characterized by polarized nucleotide composition in the two strands of DNA replication [90, 91], where the leading and lagging strands tend to be richer in keto (G and T) bases and the amino bases (C and A), respectively [92, 93]. In genomes of most of the free-living bacteria, this strand-specific nucleotide composition, as measured by their GC-skew values [93] could not impart any significant influence in the codon and/or amino acid preferences in the genes/gene-products encoded by two strands of replication. The strand-specific codon bias was observed for the first time in the intracellular pathogen *Borrelia burgdorferi *[88] and since then, more than ten bacterial and viral genomes were reported to have significant strand-specific codon bias. Strikingly enough, most of these species are obligate intracellular [94, 95]. Examples include *B. burgdorferi*, *T. pallidum* [88], *Chlamydia trachomatis* [89], *Buchnera aphidicola* [96], *Bartonella *[97], *Tropheryma whipplei* [98], *Chlamydia muridarum *[99], *Lawsonia intracellularis *[100]*,* *Ehrlichia canis *[101], adenovirus [102] etc.

Numerous hypotheses were put forward attributing the strand-specific compositional bias to the replication-induced and/or to the transcription/translation-coupled mutation/ repair asymmetry [94, 103-105]. For either kind of hypothesis, cytosine deamination of single-stranded DNA is thought to play a vital role [92, 104]. Due to the inherent asymmetry of the mechanism of DNA replication, the leading strand is exposed in the single-stranded state for a longer time than the lagging strand and hence, is more prone to cytosine deamination. During transcription, the coding strand remains in the single-stranded state for a longer time and hence, experiences more C -> T mutations. The transcription-associated asymmetries can either increase or decrease replication-associated strand asymmetries, depending on the transcription direction and the position of the gene relative to the origin of replication [105]. In most of the intracellular species displaying strong strand-specific biases, replicational and transcriptional selection are coupled together - replicational selection is responsible for the higher number of genes on the leading strand and transcriptional selection for the enrichment of highly expressed and/or essential genes on the same strand [106, 107]. Genomes of free-living bacteria have, in general, much higher plasticity, and frequent chromosomal rearrangements in these species might weaken the inter-strand compositional skews. But in intracellular microbes with reduced genomes and a protected lifestyle within the host, frequencies of chromosomal rearrangement might be too low to upset strand-specific codon biases [94]. Loss of genes for replication restart pathways in reduced genomes of intracellular microbes might also contribute to their pronounced strand-specific compositional asymmetry [108]. There are, however, some intracellular bacteria showing little or no inter-strand differences in codon bias, where genomic rearrangements are likely to occur at a higher rate despite their obligate intracellular lifestyle, as observed in *Rickettsia* [109, 110] or *Wolbachia* [111].

### d) Acquisition of Genome Islands in Organisms Having Pathogenic or Symbiotic Lifestyle

A pathogenic microbe is often distinguished from the non-pathogenic variants of the same or related species by the presence of the pathogenicity island – a flexible gene pool encoding virulence factors like toxins, adherence factors, invasion factors, secretion systems etc., clustered in a specific genomic region, the G+C-composition of which usually differ significantly from that of its core genome [57, 112]. PAIs were first described in the genomes of human pathogens of the species *Escherichia coli* [113], but with accumulation of more bacterial genome sequences, it became apparent that they represent a subclass of a more diverse group of genetic elements, designated as genomic islands (GI), found in abundance in bacterial genomes [57, 114, 115]. A GI refers to a part of a genome - usually between 10 to 200 kb in length - harbouring a number of accessory genes that might be beneficial for the host bacterium under specific environmental conditions. GIs usually differ in compositional statistics like G+C-content, cumulative GC skew, tetranucleotide frequencies, codon usage etc. from the rest of the chromosome. They are often inserted at tRNA gene loci and flanked by 16–20 bp perfect or almost perfect direct repeats [116]. They may also carry insertion elements or transposons and the same GI can occur in distantly related species. All these strongly argue in favor of horizontal acquisition of GIs by their host genomes [112, 116]. It has been suggested that GIs enable a large number of genes to be transferred and incorporated into the recipient genome that may lead to dramatic changes in the behavior of the organism resulting in “evolution in quantum leaps” [117, 118]. Evolutionary forces shaping the codon and amino acid usage in genes/gene-products of a genomic island may differ from those influencing the composition of the core genes/gene-products of its host, as demonstrated in case of the symbiotic island of the *Bradorizobium japonicum* [119].

Since acquisition of GIs often enhances the fitness of the recipient microbes facilitating microbial transmission, survival or colonization within a niche, they are also known as 'fitness islands' [120]. Fitness islands may be associated with diverse adaptive functions that contribute to different microbes' unique lifestyles. For instance, nitrogen fixation genes in *Rhizobiaceae* species are encoded by “symbiosis islands” [121], genes for phenolic compound degradation in *Pseudomonas putida* are harbored by “metabolic islands” [122], the iron-uptake ability of many pathogens are conveyed by “adaptive islands” [115] and the *mec*A-region of staphylococci that enhances survival of the carrier strains in presence of antibiotic-producing microbes in soil [123] may be termed resistance island. The same or similar GIs may exhibit distinct functionality under diverse ecological conditions or lifestyles of its host microbe. GIs in *E. coli* strains of the human gut microbiome encoding the adherence factors like P-, S-, and F1C-fimbriae [124] usually function as a saprophytic island, facilitating colonization of the gut. But under special circumstances, P-, S- or F1C-positive *E. coli* may reach the urinary tract, when the same island serves as a true pathogenicity island, helping its host microbe to emerge as a virulent strain causing infections of the bladder/kidney [125]. Similarly, GIs encoding secretion systems of type III in the virulent strains of *Salmonella* [126]*, Shigella* [127], and *Yersinia*-groups [128] or type IV in *Legionella pneumophila* [129] strains and *Helicobacter pylori* [130] are involved in the infectious process of the respective bacteria and hence, are called pathogenicity islands. But similar GIs carrying the type III system of rhizobia, or the type IV system of F plasmids act as symbiotic or ecological islands that enhance the fitness of its host microbes in their natural niche [120]. Therefore, categorization of GIs not only depends on the genetic composition of the island itself, but also on the genetic background and lifestyle of its bacterial host.

### e) Discriminatory Genome Features of Trophic Life Strategies of Marine Microbes

Marine bacteria often specialize to survive in distinct trophic habitats in the oceans - some have evolved to colonize low-nutrient (oligotrophic) environments, while others prefer to thrive in nutrient-rich (copiotrophic) sites. Comparison of genome sequences of two marine microbes, the copiotroph *Photobacterium angustum *S14 and the oligotroph *Sphingopyxis alaskensis *RB2256 as model representatives of two major classes of heterotrophic marine lifestyles, along with sequence information for 32 related microbes with well-characterized trophic lifestyles, has led to the identification of 43 genomic markers related to trophism [131]. Oligotrophs are typified by shorter genomes, fewer rRNA operons, fewer prophages, higher number of cytoplasmic and lower number of periplasmic proteins and distinct Cluster of Orthologous Groups (COG) of proteins distribution patterns. For instance, the COG categories representing defense mechanisms, cell motility, transcriptional regulators or signal transduction pathways are significantly higher in copiotrophs, while the COGs involved in transport or metabolism of lipids and secondary metabolites are typically over represented in oligotrophs. Copiotroph genomes also contain more repeats within clustered regularly interspaced short palindromic repeats (CRISPRs). There are, however, some microbes exhibiting mixed genomic traits, as exemplified by the Planctomycetes, possessing large genomes (a feature of copiotrophs), but having a single copy of the rRNA operon (a feature of oligotrophs). By creating self-organizing maps that integrated these genomic markers, Lauro *et al.* [131] could effectively distinguish microbial trophic strategies from mere knowledge of their genomic sequences.

The marine cyanobacterium *Prochlorococcus marinus *has been the first documented example of genome shrinkage along with A+T enrichment in a free-living organism [132] – an event ascribed to various factors related to their growth in oligotrophic waters [133-135], selection for metabolic economy [132, 133, 136], loss of low fitness genes [137], and smaller cell sizes [132]. Interestingly enough, it is also the first documented example of significant differences in dinucleotide abundance genome signatures across different strains of the same species [138]. On the basis of vertical niche partitioning, strains of *P. marinus* may be broadly divided into two major ecotypes: high-light-adapted (HL) ecotypes dominating the surface waters ecotype and the low-light adapted (LL) ecotype growing preferentially at depths between 80 and 200 meters. Analysis of complete genome sequences of 6 HL and 6 LL strains of *P. marinus* revealed existence of distinct dinucleotide signatures not only across the HL and LL strains, but also within LL strains of varying genome sizes and G+C-content. Analysis of synonymous codon usage profiles indicated the presence of pronounced strand-specific asymmetry in LL strains. The study [138] also delineated definite trends in amino acid usage as well as physicochemical and structural features in core proteome of different ecotypes of *Prochlorococcus* strains, which are not solely governed by their genomic G+C-bias.

### f) Distinct Genome Features in Microbes Adapted to Extreme Environments

#### i) Microorganisms Thriving at High Temperatures

Thermophiles and hyperthermphiles, *i.e.,* the organisms thriving at temperatures greater than 50° and 80° Celsius, require special adaptation strategies at genome and proteome levels to withstand extremely high temperatures. Several studies indicated a possible relationship between the optimal growth temperature (OGT) of microorganisms and their genomic base composition [46, 139, 140]. In both thermophiles and hyperthermophiles, the G+C contents of tRNA/rRNA genes exhibit strong positive correlations with their optimal growth temperature, probably to facilitate the intramolecular stabilization of RNA secondary structure at elevated temperature [47, 141, 142], but no such simple correlation exists for the genomic DNA [143]. Regression analyses of the sequence data for thermophilic, mesophilic (OGT = 20-50°C) and psychrophilic (OGT <20°C) bacteria revealed linear relationships between OGT and a combination of purine and pyrimidine dimer compositions, RR +YY – RY – YR, where R= A/G, Y = C/T), the correlation coefficient being 0.66 [144, 145]. Evaluation of the physicochemical parameters of dinucleotides suggested that such linear relationship may be attributed to distinct levels of supercoiling of DNA relevant to its thermostability [145]. It has also been demonstrated that in microbes adapted to high temperature, the purine-pyrimidine skew (R-Y)/(R+Y) correlates strongly with the location of the ORFs in two strands, so that the ORFs residing in both the direct and complementary strands, in general, tend to be purine-rich [47, 139]. While the predicted ORFs of thermophiles and hyperthermophiles are characterized by overre-presentation of purine content, the structural RNA genes of hyperthermophiles, in general, exhibit much higher G+C-content than those of the mesophiles [47].

Differences in the codon usage between thermophilic and mesophilic organisms have been described by several investigators [44-48]. Montanucci *et al.* [146] formulated a codon frequency index that could highlight robust determinants of thermostability capable of discriminating thermophilic from mesophilic genomes.

However, it is not clear yet whether the selection in favour of purine-rich coding sequences in thermophiles has its root at the nucleic acid levels or protein levels. Lobry & Chassel [45] argued that the trend for the amino-acid composition of thermophilic proteins could be under the control of a pressure at the nucleic acid level, not a selection at the protein level. It was also proposed that the selection for purine-rich mRNA sequences in thermophilic organisms may minimize unnecessary RNA-RNA interactions and prevent double-strand RNA formation within the molecule [147]. On the contrary, an analysis of 204 complete proteomes of archaea and bacteria spanning the temperature range from −10 °C to 110 °C [148] indicated that the specific amino acid composition adaptation at distinct thermal environment might be a primary factor, while the signatures at the nucleotide level, such as purine loading index, may largely be the consequences of the amino acid adaptation requirement.

#### ii) Microbes Thriving at High Salinity

Microbes thriving in hypersaline environments like the Great Salt Lake in Utah, Owens Lake in California, the Dead Sea etc. are known as halophilic (salt-loving) organisms. In order to prevent desiccation through osmotic movement of water out of their cytoplasm, halophiles employ two different strategies: the “compatible-solute” strategy, involving accumulation of osmoprotecting solutes in the cytoplasm or the salt-in strategy that relies on selective influx of inorganic ions, especially K^+^ and Cl^−^, into the cytoplasm. The compatible solute strategy does not require any significant structural adjustment in intracellular macromolecules. But in the salt-in haloadaptation, the entire intracellular protein machinery must adapt themselves to high salt level and as a consequence, taxonomically divergent halophiles show similar amino acid and other compositional biases, irrespective of their genomic GC-bias [24].

The obligatory halophiles generally contain G+C-rich genomes (well above 60%), presumably to avoid UV induced thymidine dimer formation and possible accumulation of mutations in their specialized habitat (shallow coastal lagoons), characterized by high levels of UV irradiation [149, 150]. The extreme halophilic archaeon *Haloquadratum walsbyi* is so far the only exception, with a remarkably low genomic G+C-content of 47.9% [151]. In *H. walsbyi*, the disadvantage of a low G+C-genome is thought to be partly compensated for by the presence of a relatively higher number (four copies) of photolyases [151].

All obligatory halophiles show specific dinucleotide abundance signatures, characterized by overrepresentation of CG, GA/TC and AC/GT, which may be regarded as specific genomic siganture of haloadaptation. The abundance of GA, AC and GT dinucleotides may partly be coupled with the specific amino acid requirements at the protein level, while the abundance of CG increases the propensity to transition from B-DNA to a Z-DNA conformation that is stabilized at high salt concentrations [152]. The requirement for Asp, Glu, Thr and Val residues in halophilic protein sequences increases frequencies of GA, AC and GT dinucleotides at the first and second codon positions of the genomic DNA. The pattern of synonymous codon usage in halophiles has also shown to be significantly different from that in non-halophiles.

## CONCLUDING REMARKS

The genomic architecture of a microbe often bears the signatures not only of its phylogenetic position, but also of the kind of lifestyle to which it is adapted. Two taxonomically, spatially and temporally distant microbial populations may acquire similar genomic traits, if they intend to flourish at similar ecological niches; while two closely related microbes, when acclimatized to differential environmental conditions, may undergo adaptive radiations through selection of conspicuous genomic traits. Niche-specific genome signatures may include but are not restricted to the sequence features like base composition, GC-skew, purine-pyrimidine ratio, dinucleotide abundance, codon bias, and oligonucleotide composition, presence of specific gene-families, horizontal acquisition of genome islands and the processes of genome shrinkage/expansion. The remarkable diversity in the niche-specific signature features, characterized so far, underscores the evolutionary plasticity of the microbial genomes and there are reasons to believe that this represents only the tip of an iceberg - with most of the signature features remained to be explored and unveiled yet. With ever-increasing number of microbial genome sequences in the public domain, one would expect revelation of many more novel niche-specific genome signatures in microbes adapted to different specialized lifestyles or extreme ecological niches. Such signatures, if properly interpreted, may not only offer insight into the molecular strategies of niche specialization in microorganisms, but may also have far-reaching implications of metagenomic and biotechnological perspective.
